# Production and Characterization of Xanthan Gum from Low-Quality Dates of Different Cultivars as a Fermentation Substrate

**DOI:** 10.3390/polym18172074

**Published:** 2026-08-26

**Authors:** Reem A. Altwijri, Abdellatif A. Mohamed, Suleiman A. Althawab, Hany M. Yehia, Abdulrahman Alahmed, Shahzad Hussain

**Affiliations:** Department of Food Science and Nutrition, King Saud University, Riyadh 11451, Saudi Arabia; 443203579@student.ksu.edu.sa (R.A.A.); abdmohamed@ksu.edu.sa (A.A.M.); salthawab@ksu.edu.sa (S.A.A.); hanyehia@ksu.edu.sa (H.M.Y.); abalahmed@ksu.edu.sa (A.A.)

**Keywords:** dates, sugars, xanthan, rheology, thermal, yield, activation energy

## Abstract

Approximately 5–33% of the dates growing in Saudi Arabia are downgraded to low-quality fruit that either goes to waste or is used to make animal feed, which is considered a potential feedstock abundant in sugar. By a thorough comparative study, this study evaluated the up-cycling of low-quality Saudi dates (Wannana, Shagra, Sabbaka, Barhi, Saqai, Khalas and Sukkari) as fermentation substrates for xanthan gum production by *Xanthomonas campestris*. Pure glucose, pure sucrose, and a commercial standard were used as a baseline. The sole carbon source was date juice (≈12.5–17 °Brix) in a batch aerobic fermentation conducted at the standard conditions of temperature (30 °C), speed 180 rpm, and time (120 h). The xanthan gum was quantified and tested for its properties like functional groups (FTIR), color, thermal behavior (TGA and DSC), and rheology in the form of both steady- and dynamic-shear rheology. Xanthan gum was produced in the range 5.60–7.77 g L^−1^ by the date-based substrates with Barhi (7.77 g L^−1^) and Saqai (7.58 g L^−1^) surpassing those of the glucose (6.68 g L^−1^) and sucrose (6.23 g L^−1^) controls. FTIR spectra of date-derived gums were almost identical to that of the commercial standard, indicating that their functional groups and the primary structure were very similar. In addition, the date-derived powders were darker and yellower (L* 61.09, 71.00; whiteness index 54.92, 63.34) than the commercial gum (L* 84.71; whiteness index 78.19). This is likely attributed to the presence of residue date pigmentation and products of Maillard and caramelization. Thermogravimetric analyses revealed that the breakdown of materials occurred in two stages, of which the char residue of the date-derived gums was much higher for those degraded at 500 °C (48.93, 54.05%) versus that of the commercial reference (33.49%), which is to be interpreted as a better ability of the former to resist thermal degradation. All solutions acted as pseudoplastic, shear-thinning liquids (flow behavior index *n* < 1); the consistency coefficient (*K*) rose with concentration and fell with temperature. The activation energy varied between 9.98 kJ mol^−1^ (commercial) and 29.39 kJ mol^−1^ (Saqai). Overall, low-quality Saudi dates can be considered a technically and economically viable, sustainable, and low-cost carbon substrate suitable for upcycling to produce xanthan gum, which is safe for use as a food additive.

## 1. Introduction

Upcycling, a concept increasingly used in the food business, denotes the process of transforming byproducts or waste materials into new goods of superior value, rather than recycling them into materials of equivalent or worse quality [1]. The primary distinction between upcycling and recycling resides in their capacity to enhance value. Recycling emphasizes the transformation of waste into raw materials or products of equivalent or lower value. This is more advantageous than conventional materials, as it not only minimizes waste but also creates new market opportunities, particularly in sectors such as food production, where waste can be transformed into high-value materials or functional products [2].

Originating in the Arabian Peninsula, the date palm (*Phoenix dactylifera* L.) is the oldest tree cultivated, with a history of consumption spanning over 7000 years. It is a member of the Arecaceae (Palmae) family [3]. Dates are the principal fruit grown in the Kingdom of Saudi Arabia. Dates contain carbohydrates (glucose, fructose, and sucrose) [4,5] as well as other components such as minerals, proteins, fats, and vitamins, all of which are necessary components of a well-balanced diet [6,7]. Dates have traditionally been an essential component of the bulk of Middle Eastern diets [8].

Date production is vital to the economies of numerous countries, especially in the Middle East and North Africa. However, not all dates produced are of outstanding quality for direct consumption. Approximately 50% of cultivated dates produced in Iran are deemed low-quality and are typically utilized as animal feed or dumped as garbage, resulting in significant environmental and economic repercussions [9], while another study reported that low-quality dates waste accounts for 10–15% of the total production [10]. El-Habbab et al. [11] reported losses of 5–33% across different varieties of Saudi dates (Khalas, Shishi, Sukkary, Segae, Shagra, Berhi, and Ajweh) in various regions of Saudi Arabia.

Recently, natural polysaccharides have gained popularity among companies and consumers for their biodegradability, nontoxicity, and biocompatibility compared to synthetic or chemical polymers [12]. Microbially derived polysaccharides are safe, biodegradable, and environmentally benign. They also maintain their activity in the presence of extremes in pH, salinity, and temperature [13]. There have also been reports of genetically altered microorganisms being used to produce novel goods with unique features [14].

Industrial production of microbial polysaccharides such as pullulan, curdlan, scleroglucan, dextran, and xanthan occurs under controlled bio-fermentation conditions for commercial use [15,16,17,18,19]. Xanthan gum, a microbial polysaccharide produced by *Xanthomonas campestris* thru fermentation, is a key biopolymer used in the food industry due to its thickening and stabilizing abilities [20,21,22,23]. This polymer exhibits unique gel and viscous solution formation during fermentation [24]. The high molecular weight, rheological properties, and water-solubility of xanthan gum make it useful in various industries, including food, pharmaceutical, cosmetic, and petroleum and oil [23,25,26].

Traditionally, xanthan gum manufacturing relies on glucose or sucrose as substrates, which will drastically increase its manufacturing cost [21]. The excessive use of xanthan gum in the food industry, combined with growing pressure to lower production costs, has sparked interest in alternative, low-cost substrates, including agricultural waste. In this context, low-quality dates, which can be wealthy in sugars, constitute a valuable resource for xanthan gum manufacturing through microbial fermentation [22].

Therefore, the problem of overuse of low-quality dates can be solved by upcycling. This is the process of turning waste materials into higher-value products. In the case of low-quality dates, it is used as a fermentation substrate for gum production. Therefore, it creates a sustainable and economically viable alternative to conventional substrates [26,27]. This upcycling method aligns with global sustainability goals, reduces the environmental impact of agricultural waste and provides a cost-effective method of Xanthan gum production [28]. The aim of this research is to explore the potential of upcycling low-quality Saudi dates into xanthan gum. The specific objectives of the current work were to explore the use of low-quality dates as a cost-effective substrate whilst systematically correlating the composition of the substrate with yield, physiochemical properties and rheological nature of the resultant polymer to compare it with commercial xanthan gum. This will help to reduce waste and produce commercially valuable products. The production and characterization of locally produced xanthan gum from food waste will open the door to its commercial production and application in the local and international food, pharmaceutical, petroleum, and other sectors.

## 2. Materials and Methods

### 2.1. Materials

This research utilized low-quality dates from seven selected varieties (Wannana, Shagra, Sabbaka, Barhi, Saqai, Khalas and Sukkari) as substrates (as carbon sources) for xanthan gum production. The low-grade raw dates were collected from a local farm in Al-Qassim. The low-quality dates, which are typically discarded or used as animal feed due to their poor quality, are ideal candidates for upcycling in this fermentation study. *Xanthomonas campestris* (MTCC, 2286) obtained from Microbial type Culture Collection and Gene Bank of Institute of Microbial Technology, Chandigarh, India was used to ferment the substrate for the xanthan gum production. Pure sucrose and glucose were also used as control substrates.

### 2.2. Substrate Preparation, Microorganism and Culture Media

After being sorted, the dates were pitted and the fleshes were washed and ground into a slurry using of a mechanical grinder. The date juice was prepared by adding water to the date paste at a ratio of (3:1, *v*/*w*). The date paste–water mixture was gently boiled for 15 min with continuous stirring. About 2.5 L of date juice were obtained from 1 kg of date. The soluble solids content (°Brix) of the date juice was recorded between 12 and 18 °Brix, which was then used to adjust the sugar concentration in the production media. It was determined at 25 °C using a handheld refractometer (Erma, Tokyo, Model No. 50010).

The preparation of the inoculum was performed by the transfer of the microorganism from the stock solution to the Yeast Mold agar plates (YM agar) and its subsequent incubation for 48 h at 30 °C. A loopful of cells from the (YM) plates was then transferred to a 250 mL conical flask containing 100 mL of the sterile YM medium and incubated for 24 h at 30 °C and 180 rpm. This was ultimately used as the inoculum. Fermentation was carried out in 500 mL Erlenmeyer flasks, each of which contained 250 mL of the sterile production medium. The medium was inoculated with 5% (*v*/*v*) of the *X. campestris* culture.

The medium used for the growth and maintenance (YM agar) contained (g/L): glucose, 46.6; peptone, 5; yeast extract, 3; and agar, 17 (pH 7). Bacterial cells in agar slants were incubated for 48 h at 30 °C and then stored at 4 °C for further use. The medium used for xanthan gum production contained (g/L): sugar,12 g (either adjusted from date juice or sucrose/glucose); KH_2_PO_4_, 5; MgSO4, 0.2; (NH4)_2_SO_4_, 2.7; citric acid, 2; H_3_BO_3_, 0.006, ZnCl_2_, 0.006; FeCl3, 0.0024 and CaCO_3_, 0.02. The pH was measured at 20 °C using a Corning Model5 pH meter (Corning Scientific Instruments, New York, NY, USA) and adjusted to 7 by the addition of NaOH (1 M). The solutions were sterilized for 20 min at 121 °C.

### 2.3. Xanthan Gum Production

A modified method followed by Salah et al. [29] was used to optimize the fermentation procedures. Xanthan was produced (3 independent batches) using batch aerobic fermentation on an orbital shaker at 30 °C and 180 rpm for 120 h. Once the fermentation was complete, the culture broth was subjected to a series of purification steps to isolate the xanthan gum. The fermentation broth was centrifuged at 10,000 rpm for 20 min to remove microbial cells and other solid impurities. The clear supernatant containing the xanthan gum was mixed with absolute ethanol at a 1:2 ratio (supernatant to ethanol) to precipitate the xanthan gum. The mixture was then left to stand for several hours to allow complete precipitation. The precipitated xanthan gum was filtered and washed with additional ethanol to remove residual impurities. The produced xanthan gum was air-dried at 40 °C, and the dried product was ground into a fine powder and stored in airtight jars for further analysis [30].

### 2.4. Characterization of Xanthan Gum

#### 2.4.1. Extraction Yield

The yield of the gums was calculated as the dry weight of the gum powder relative to the volume of the substrate and expressed as g/L.

#### 2.4.2. Fourier-Transform Infrared Spectroscopy (FTIR)

The FTIR spectrum of the gums was generated using a Bruker ATR–FTIR Spectrometer. The spectra were obtained between 600 and 4000 cm^−1^. The data was plotted as wavelength vs transmittance% using Sigmaplot 10.0 software.

#### 2.4.3. Color Analysis

Color was measured using a SATAKE color grader (NCGA, Higashi-Hiroshima, Japan), using the D65 color source. Color values like L*(Lightness), a* (Redness) and b* (Yellowness) of gum samples were determined. L* = 0 or 100 indicate black and white respectively; a* the axis of chromaticity between green (−) to red (+), and b* the axis between blue (−) to yellow (+).

#### 2.4.4. Differential Scanning Calorimeter (DSC) Conformational Changes

Thermal analysis was carried out using differential scanning Calorimeter (Q 2000, TA instruments, New Castle, DE, USA). The enthalpies associated with conformational changes were determined for different xanthan gum powders during heating ramp of 10 °C/min from 25 to 225 °C.

#### 2.4.5. Thermal Stability of Gum Powders

Thermal stability of the gum powders (5–10 mg) was analyzed using a thermogravimetric analysis (TGA) (TA Instruments, New Castle, DE, USA) using 30 °C heating rate in the temperature range between 30 and 500 °C using the method followed by [31].

#### 2.4.6. Rheological Measurements

##### Steady Shear Rheology

Hydrocolloid solutions at different concentrations (0.25, 0.50, 0.75, and 1%) were prepared by adding the powdered gum in distilled water and then vigorously stirring at 60–80 °C. The solutions were stirred for 2–3 h to ensure complete solubilization. Volume adjustment was made after cooling the solutions to room temperature. The gum solutions were subjected to steady shear behaviors (shear rate vs. shear stress) at different concentrations and temperatures (20, 30, 40 °C) at varying shear rates from 1 to 100/s.

Equation (1) was used to fit the data obtained from all the measurements to the power law model.

(1) $$\tau =k\Upsilon^{n}$$

where τ = shear stress (10 dyn/cm^2^), *k* = consistency coefficient (Pa s^n^), γ = shear rate (s^−1^), and *n* = flow behavior index (dimensionless).

Equation (2) was used to determine the temperature dependency of the samples. The viscosity data obtained at different temperatures was fitted in Equation (2) according to the method followed by Mohamed et al. [32].

lnK = lnK_0_ + (Ea/R)(1/T) (2)

R = Universal gas constant (8.314 J mol^−1^ K^−1^)

T = absolute temperature (K)

K (Pa s^n^) = power law consistency index representing the consistency or apparent viscosity of the xanthan solutions

K_0_ (Pa s^n^) = is the frequency factor at a reference temperature (20, 30 and 40 °C), and is obtained from the intercept of the Arrhenius plot

*Ea* = Energy of activation

##### Dynamic Rheology

The dynamic rheological testing was performed using a Dynamic Hybrid Rheometer (DHR) (TA Instruments, New Castile, PA, USA). Frequency sweep testing was done at an angular frequency ranging from 0.1 to 50 rad/s and a strain of 5%. The energy contained in the material and recovered from each cycle was measured by the storage dynamic modulus (G′), whereas the energy lost (dissipated) every cycle of sinusoidal deformation was calculated as the loss modulus (G″). Tan δ defines the ratio of energy stored to lost energy for each cycle.

#### 2.4.7. Statistical Analysis

The Data was collected in triplicate and reported in the tables as means ± sd. The data was analyzed using the one-way analysis of variance (ANOVA) technique. The Duncan’s Multiple Range (DMR) test at *p* ≤ 0.05 was employed to compare means using SPSS (PASW^®^ Statistics 18) software.

## 3. Results and Discussion

### 3.1. Composition of the Date Juice Substrates

The soluble-solids content and sugar profile of the date juices prepared from the seven cultivars are summarized in Table 1. The total soluble solids ranged from 12.5 °Brix for Khalas to 17.0 °Brix for Sukkari. This wide range reflects cultivar-dependent differences in ripening stage, moisture content, and flesh-to-water extraction behavior during juice preparation. Despite this spread in °Brix, the fermentable-sugar content of the juices was relatively uniform. Glucose and fructose together account for the bulk of the dissolved carbohydrates in every cultivar. Glucose contents ranged from 5.00% (Sukkari) to 8.84% (Wannana), while fructose contents varied between 4.69% (Sukkari) and 8.83% (Sabbaka).

However, the near-equimolar distribution of glucose and fructose in six of the seven cultivars (Barhi, Saqai, Wannana, Sabbaka, Shagra and Khalas), coupled with negligible sucrose (0.00–0.12%), is considerable. This ≈ 1:1 glucose-to-fructose ratio is the fingerprint of invert sugar and indicates that the sucrose present natural in the fruit had been completely hydrolysed into its constituent monosaccharides by endogenous invertase during the advanced (tamr) stage of ripening [6,8]. Sukkari was the exception; it kept 6.91% sucrose alongside lower glucose (5.00%) and fructose (4.69%) fractions, a compositional signature consistent with the soft, semi-dry character of this cultivar, in which sucrose inversion is less complete.

From a fermentation standpoint, this composition is highly favorable. *X. campestris* preferentially and rapidly assimilates glucose, and to a lesser extent fructose, for growth and xanthan biosynthesis. Therefore, the predominance of readily fermentable monosaccharides in the date juices minimizes the need for prior hydrolysis and renders these substrates directly usable as carbon sources [22,29]. On the other hand, with regard to simple sugars, date juice is also a natural reservoir of minerals, nitrogenous compounds, and vitamins [6,7]. It can act as a supplementary growth factor during fermentation. Collectively, these findings confirm that low-quality dates, irrespective of cultivar, furnish a sugar-rich medium well suited to microbial xanthan production.

### 3.2. Production Yield and Composition of the Recovered Solids

The raw yeild representing the total solids including protein residues, ash and mositure, polymer obtained from the different substrates after 120 h of fermentation are presented in Table 2. The range of ash content was from 12.60 ± 0.13 to 16.51 ± 0.85%, while protein content varied from 0.68 ± 0.06 to 1.54% for all lab-grown samples. With respect to commercial xanthan gum (7.52 ± 0.77% ash and 0.17 ± 0.03% protein), these higher contents indicate that small quantities of inorganic and non-polysaccharide components could not be completely eliminated after recovery. It has been theorized that residual ionic species may alter xanthan chain conformation and thus affect rheological properties, while relatively low protein levels are unlikely to play a significant role [33]. The rehogical data presented in later sections also suggest that samples with higher ash value reprented relatively low consistancy coefficients. However, as individual minerals and protein fractions were not characterized, these specific effects on rheological properties cannot be determined unequivocally.

Yields from the date-based media ranged from 5.60 g L^−1^ (Khalas) to 7.77 g L^−1^ (Barhi). The substrates were ranked as follows: Barhi > Saqai (7.58) > Sukkari (7.25) > Sabbaka (7.22) > Shagra (6.97) > Wannana (6.45) > Khalas (5.60). It is noted that five of the seven date cultivars matched or surpassed the pure-sugar controls, glucose (6.68 g L^−1^) and sucrose (6.23 g L^−1^). Specifically, Barhi and Saqai out-yield both reference substrates by a clear margin. This finding demonstrates that a low-value waste stream can rival, and even exceed, the refined sugars conventionally used for industrial xanthan production.

The competitive performance of the date media is attributable to their compositional richness. Whereas glucose and sucrose supply carbon alone, date juice concurrently provides fermentable monosaccharides together with minerals, free amino nitrogen, and vitamins that act as growth promoters for *X. campestris*. All these processes support denser biomass and more efficient exopolysaccharide biosynthesis [6,26]. The highest-yielding cultivars, Barhi and Saqai, were also among those with the greatest combined glucose-plus-fructose content, which is consistent with the well-established dependence of xanthan productivity on the availability of readily assimilable carbon [14,34].

Since the sugar concentration of date juices was standardized in the media, the comparatively low yield from Khalas juice (5.60 g L^−1^) may be due to the consequence of intrinsic inhibition caused by the presence of nitrogenous compounds, minerals, and other minor nutrients, which may affect bacterial growth and xanthan biosynthesis. The yields recorded in our study are in broad agreement with, and in several instances exceed, those previously reported for xanthan gum produced from date syrup and date by-products [9,22,29]. At the same time, they are comparable with values obtained from other agro-industrial substrates such as grape and olive pomace, kitchen waste, and confectionery wastewater [28,35,36,37]. These results confirm that low-quality Saudi dates constitute an efficient and economically attractive substrate for xanthan gum fermentation. Since the concentration of residual sugars (glucose, fructose, sucrose) after the fermentation were not determined, the reported xanthan values are indicative of product yield rather than conversion efficiency. This is the limitation of the current research; therefore, future studies should assess sugar consumption to more accurately gauge substrate utilization.

### 3.3. Color Analysis of Xanthan Gum Powders

The color parameters (L*, a*, b*), total color difference (ΔE), and whiteness index (WI) of the xanthan powders are reported in Table 3. A statistically significant variation was observed between the gums fermented on date juice and the pure sugar and commercial references. The commercial standard was the brightest and most neutral sample (L* 84.71, a* −0.02, b* 15.55) and possessed the highest whiteness index (WI 78.19), followed by the sucrose-derived gum (L* 82.81, WI 69.17). By contrast, all date-derived powders were appreciably darker (L* 61.09–71.00), more reddish (a* 4.00–7.86) and more yellow (b* 19.13–25.44), with correspondingly lower whiteness indices (WI 54.92–63.34) and large total color differences (ΔE 17.37–25.00) relative to the reference.

Among the date cultivars, Sukkari yielded the lightest powder (L* 71.00), whereas Shagra (L* 61.09) and Saqai (L* 62.30) produced the darkest. Khalas exhibited the most intense chromaticity, recording the highest a* (7.86) and b* (25.44) values and the most saturated red-yellow hue. The pronounced yellow-brown coloration of the date-based gums may be attributable to two factors: first, the co-precipitation of native date pigments (carotenoids, polyphenols, and their oxidation products) that are extracted into the juice and carried through fermentation into the recovered biopolymer. Second, the formation of colored Maillard and caramelisation products generated from the abundant reducing sugars during the boiling step of juice preparation and subsequent processing [6,10]. The comparatively whiter sucrose-derived gum supports this interpretation, since sucrose is a non-reducing disaccharide and is therefore far less prone to Maillard browning than the reducing-sugar-rich date juices or the glucose control (L* 69.93). The other factors that relate to potential binding of substrate-derived pigments with xanthan polymer matrix could also contribute. Since pigment–polymer interactions and measurements of trace metal ions were not assessed in this study, it is unclear how much these factors contribute to the final color. Overall, more studies with adequate control experiments including pigment removal before fermentation and elemental analysis are needed to clarify the relative roles of these mechanisms in color development.

It should be emphasized that this coloration is a purely aesthetic and purification-related attribute and does not reflect any change in the chemical identity of the polymer. Comparable pigment carry-over has been reported for xanthan gum synthesized on other colored agro-industrial substrates, where the depth of color is governed largely by the extent of downstream purification [37,38]. For applications demanding a white product, the colour of the date-derived gums could be readily reduced by an additional decolourisation step, like activated-carbon treatment or repeated ethanol washing, without compromising the functional properties established in the following sections.

### 3.4. Fourier Transform Infrared Spectroscopy (FTIR) Analysis

**Figure 1:**
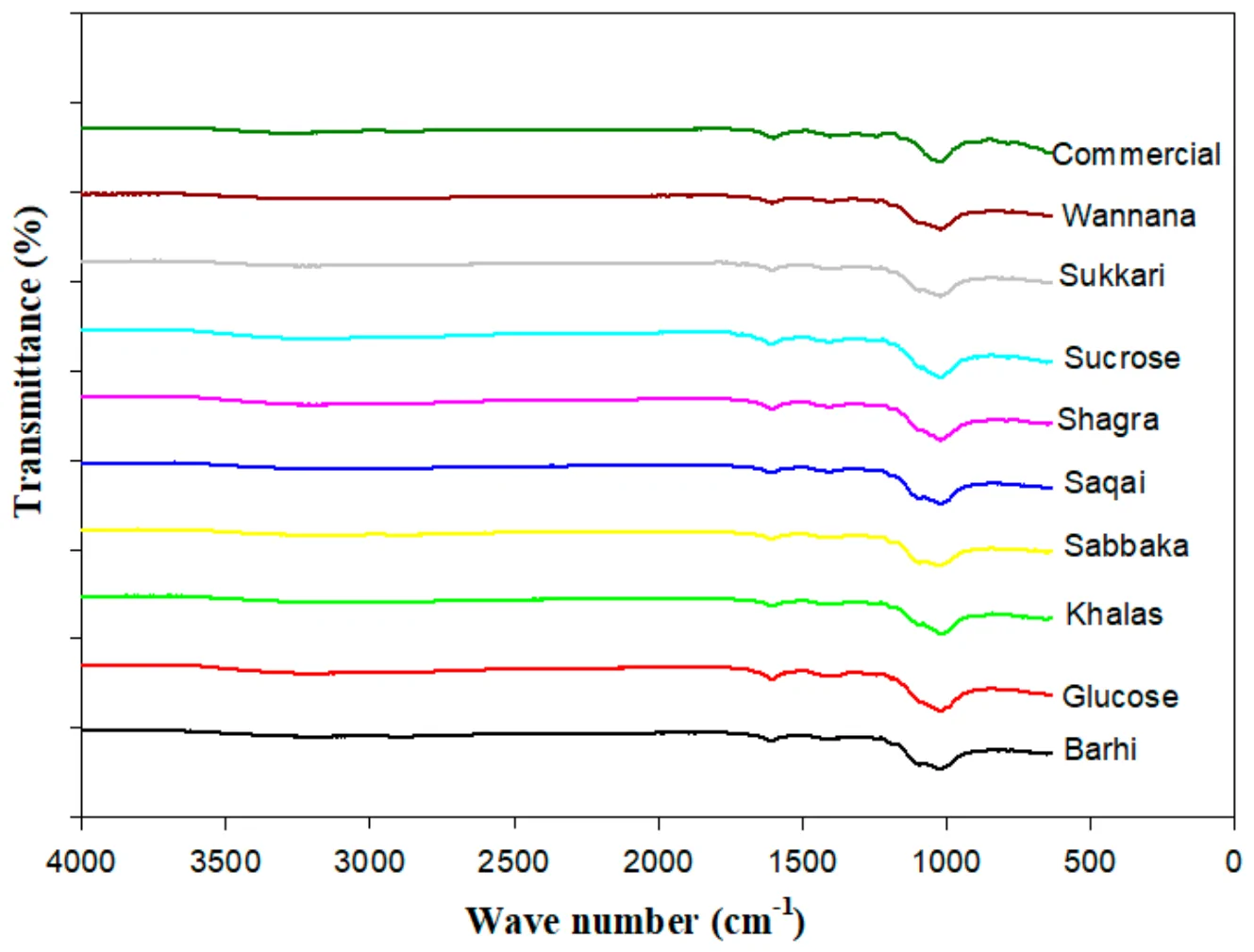
FTIR spectra of the xanthan gum powder.

The Fourier-transform infrared (FTIR) spectroscopic evaluation of xanthan gum powders produced from different substrates of date cultivars, pure sugars (glucose and sucrose) and the commercial standard are presented in Table 4 and Figure 1. It can be observed that all the samples had very similar spectral patterns, which completely confirms the existence of the functional groups specific to xanthan gum macromolecules. The spectra have shown the absorption bands at 782–789 cm^−1^ (related to vibrations of pyranose ring), 1014–1019 cm^−1^ (associated with C–O–C, C–O stretching attributed to glycosidic bonds), 1240–1405 cm^−1^ (characteristic symmetric stretching of COO^−^ moieties along with C–H bending and C-O vibrations), 1600–1604 cm^−1^ (asymmetric stretch for COO- groups), 1714–1715 cm^−1^ (C=O stretching of acetyl or pyruvate functional groups), 2866–2901 cm^−1^ (C-H stretching vibrations of methylene groups), and 3192–3234 cm^−1^ (O-H stretch vibration associated with hydroxyl groups). Importantly, this particular pattern of spectral features is fully consistent with the previously established FTIR profile characteristic for xanthan gum as extensively reported in the existing literature [39,40].

A closer examination of the specific spectral bands reveals that, within the region 3192–3234 cm^−1^, a very broad and clear absorption band is mainly due to overlapping O–H stretching vibrations. These particular vibrations are primarily attributed to the presence of numerous hydroxyl groups along with the networks of intermolecular and intramolecular hydrogen bonding that are inherent in xanthan polysaccharide matrix [41]. In addition, the distinct absorption band observed at 2866–2901 cm^−1^ corresponds to symmetric stretching vibrations of aliphatic C–H bonds (probably in connection with the methyl and methylene groups present in both the main structural backbone of xanthan biopolymer and side chains branched from it) [41,42]. A very strong and well-defined absorption band at exactly 1714–1715 cm^−1^ is assigned to the stretch vibration of carbonyl (C=O) groups present in all samples. These particular groups originate directly from the acetyl and pyruvate ester substituents and have a critical effect on the rheological behavior of the xanthan gum [43]. The band found between 1600 and 1604 cm^−1^ corresponds directly to the asymmetric stretching vibration of carboxylate (COO^−^) groups from glucuronic acid residues, which correspondingly confirm the inherent anionic character of gum macromolecule structure. The moderately broad absorption region between the wavenumbers of 1240 and 1405 cm^−1^ is associated with a complex mixture of symmetric COO^−^ stretching, C–H bending vibrations, and C–O stretching vibrations linked to the acetyl groups found on the polymer [43,44]. In addition, the absorption band observed at 1014–1019 cm^−1^ was reasonably connected with the C–O–C stretching vibrations of glycosidic linkages and pyranose rings, representing a spectroscopic integrity of the polysaccharide backbone [45]. Also, the extremely characteristic and distinct absorption band appearing at the lower frequency region of 782–789 cm^−1^ corresponds directly to the complex vibrational modes from the pyranose ring deep in the highly specific spectral fingerprint area, further providing strong and clear additional evidence of the presence of the characteristic molecular structure for synthetized xanthan gum [35,44].

Certainly, the observations from this spectroscopic examination are the uniformity of all FTIR spectra obtained from our entire experimental sample set. Regardless of whether the xanthan gum biopolymer was biosynthetically produced using a diverse range of date cultivars (namely, Sukkari, Barhi, Saqai, Wannana, Sabbaka, Shagra and Khalas) or produced from simple sugars like glucose and sucrose or even a direct comparison to an industrial standardized xanthan gum, the intrinsic characteristic absorption bands always appeared at very similar and highly reproducible wavenumbers with only minor (<±1–4 cm^−1^) significant spectral shifts. Therefore, one can confidently conclude that distinct date cultivars’ diverse extracts are able to produce xanthan gum which has the same basic chemical structure as the commercially available xanthan gum produced by using highly purified industrial sugars and other common commercial sources. These detailed and very encouraging analytical results ultimately emphasize the extraordinary biological stability, as well as robustness, of the microbial xanthan biosynthetic pathway, hence providing strong empirical support for future utilization of date juice obtained from various cultivars as an efficient, sustainable, inexpensive and risk-free alternative carbon source for the large-scale industrial production of xanthan gum without endangering its vital molecular compositional or functional properties.

### 3.5. Thermogravimetric Analysis of the Xanthan Samples

**Figure 2:**
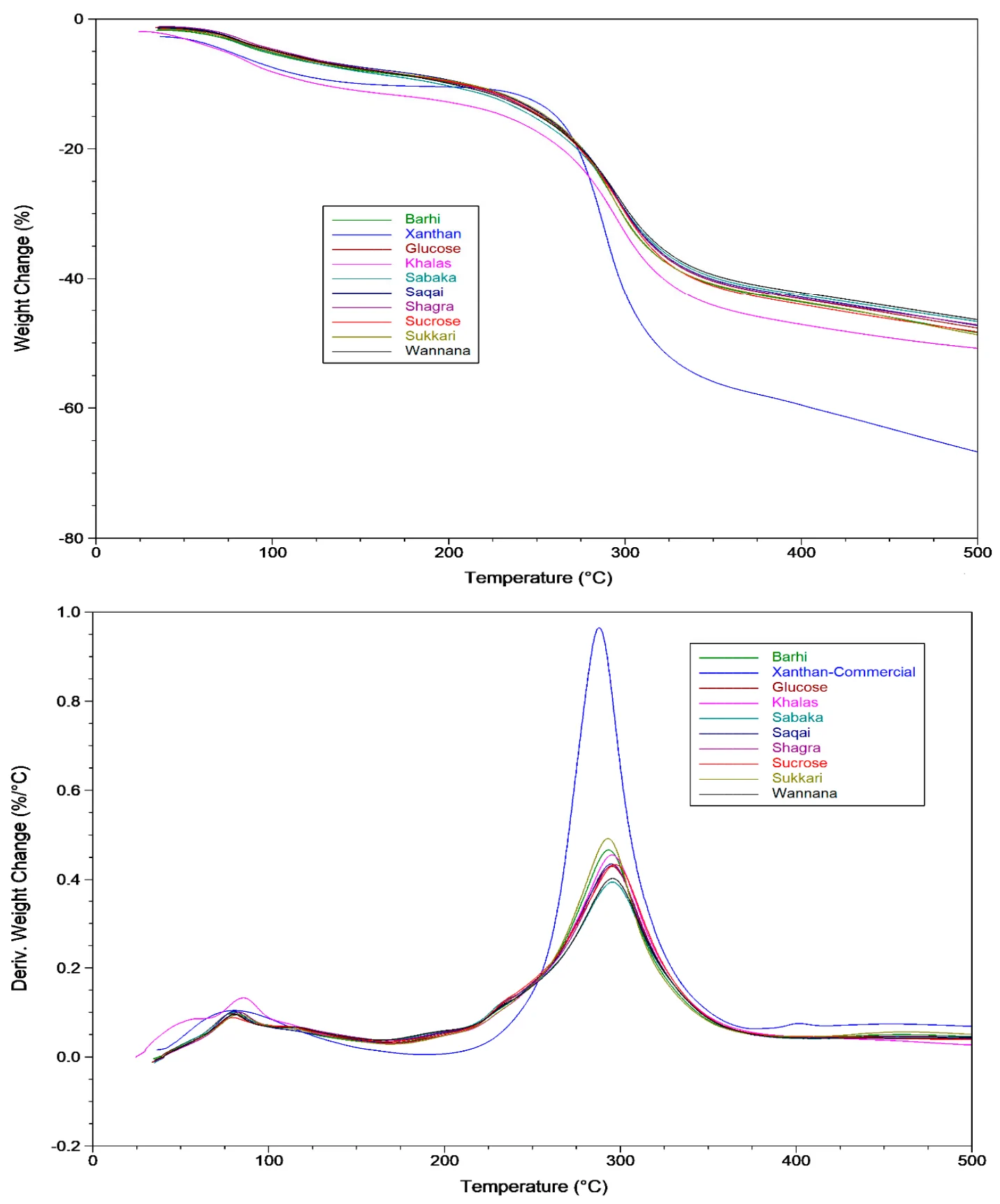
TGA and dTGA curves of different xanthan samples.

It is evident from the TGA data (Table 5, Figure 2) that all the samples including commercial xanthan followed two stage degradation patterns. All the date-based substrates yielded xanthan gum with a similar pattern of thermal stability. The degradation pattern followed by samples in the current study is the same as that reported by previous studies on xanthan [46,47,48]. Stage 1 (35–170 °C) corresponds to moisture evaporating with low mass loss between 7.61 and 11.17% for Barhi/Wannana and Khalas samples, respectively. The DTG maximum was recorded between 76.04 and 83.02 °C among different samples for the peak 1. The weight loss during stage 1 is related to the evaporation of water molecules (physically adsorbed or bound). The relatively low moisture evaporation from the samples could be linked to the presence of abundant hydroxyl and carboxyl groups making hydrogen bonds with the water molecules and disallowing them to be freely released during the first stage [46,49,50].

The second stage (160–408 °C), corresponding to the DTG peaks between 288.36 and 297.90 °C, showed that the major thermal decomposition of the polymer occurred during this temperature range. Second-stage weight loss (29.08–33.92%) varied little between date-derived samples, showing that the fermentation substrate had a minor effect on decomposition mechanisms. This second stage decomposition is linked with the cleavage of glycosidic linkages, depolymerization of the backbones, decomposition of the acetyl and pyruvate substitutes and finally decarboxylation of the glucuronic residues under the nitrogen environment with the final residue of carbonaceous structures [46,49,51]. The weight loss during the second decomposition was highest in commercial samples (42.89%). The xanthan samples produced from different date varieties presented the higher levels of residues (48.93–54.05%) at 500 °C, presenting a greater thermal resistance under nitrogen. The higher residues in such samples indicate that they possess almost double the quantity of ashes and proteins compared to the commercial sample, as presented in Table 2. The observations were further supported with DTG curves. Date-derived xanthan displayed broader peaks around 293–297 °C (characteristic of gradual, orderly degradation), while the commercial xanthan gum sample represented a sharp peak (288 °C) suggestive of quick decomposition. In sum, xanthan gums derived from date juice showed a thermal stability considerably higher than that from commercial xanthan, suggesting their potential of utilization in high temperature processing, e.g, extrusion, baking and spray drying where thermal stability of hydrocolloids is preferred.

### 3.6. Differential Scanning Colorimeter (DSC) Analysis

**Figure 3:**
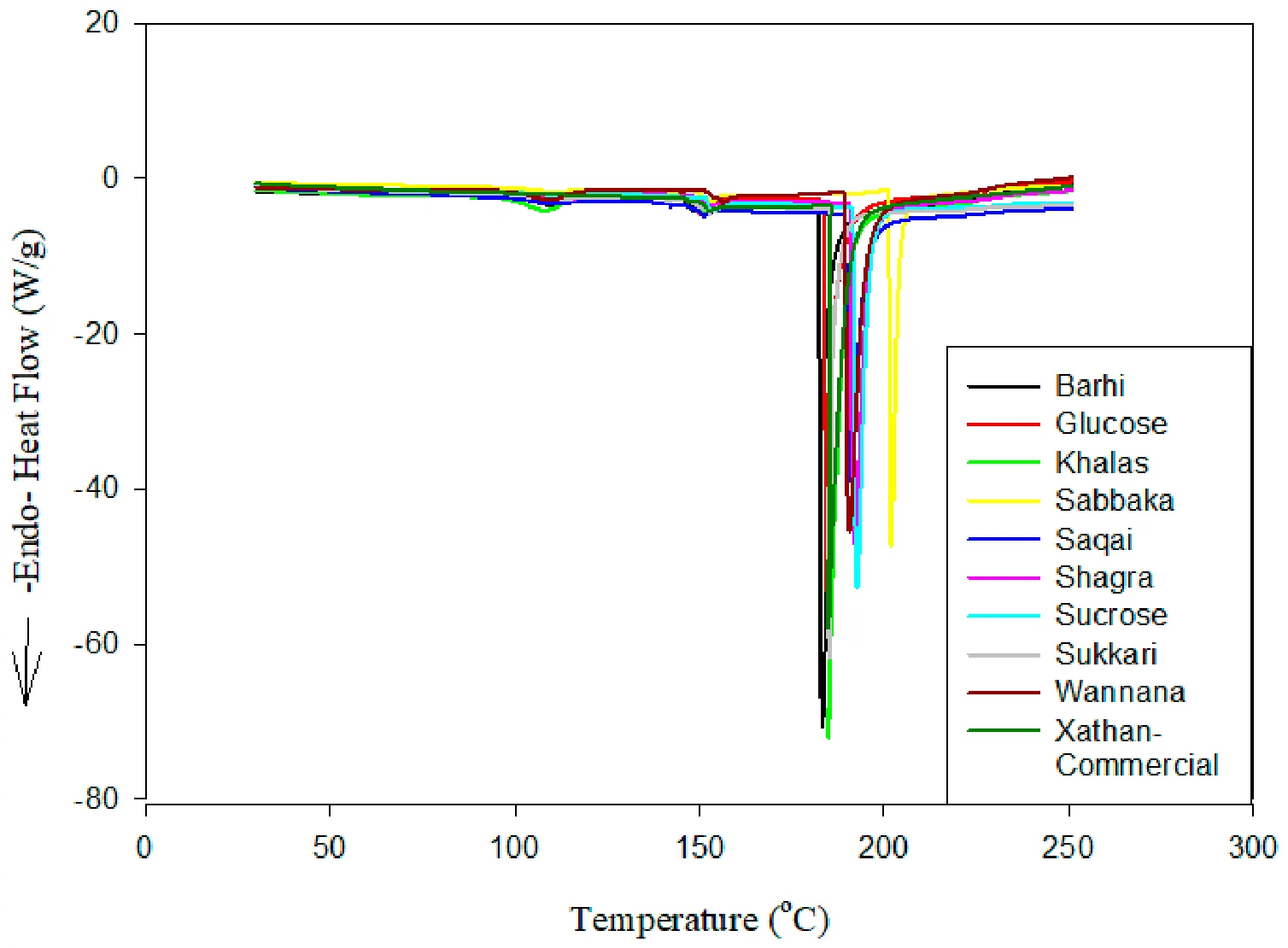
DSC thermograms of different xanthan samples 3.7. Rheological behavior of xanthan gum samples.

The data pertaining to the DSC characterization of different xanthan samples is presented in Table 6 and Figure 3. It is evident from the results that dried xanthan samples have shown three endothermic and 1 glass transition event between the studied temperature range (30–250 °C). The peaks were identified as moisture evaporation, glass transition, enthalpic relaxation and melting. The DSC thermograms of the xanthan gum produced in the laboratory were significantly different to that of commercial. The initial endothermic peaks representing moisture loss were endothermic corresponding to the moisture present in the samples. The first peak in the thermal analysis of the lab sample was seen at 62.82–65.88 °C. The results also indicate that relatively low values (0.65–1.92 J/g) were required to remove the associated moisture content from the samples. The peak observed for the commercial samples was relatively late and in the middle of two peaks as represented by lab grown samples. The second larger peak maxed around 108.25–111.97 °C (ΔH 6.55–10.99 J/g) is indicative that strongly bonded water exists throughout the polymer network formed during synthesis. The commercial sample exhibits no such secondary peak and a corresponding low-enthalpy event happened at 78.41 °C, with ΔH 1.55 J g^−1^. Zohuriaan and Shokrolahi [47] reported the endothermic event in pure xanthan gum around 81 °C, while [52] recorded the event at 76.4 °C in commercial samples. The study by Fenin et al. [53] reported the peak at 108 °C for natural, non-modified xanthan samples. This difference occurs because prolonged industrial processing removes impurities, compounds that help retain water. Xanthan produced in the lab may contain residual salts or fermentation components, which are probably still present because of incomplete purification that enhances water binding through ionic and hydrogen interactions. The values of glass transition (Tg) were between 137.28 and 145.26 °C, with maximum in commercial xanthan (145.26 °C), followed by Wannana (144.96 °C). Tg is the polymer stability marker that represents its transition from a glassy to a rubbery state [54]. A higher Tg indicates less mobility and stronger intermolecular attraction while a lower Tg indicates plasticization [55]. The consensus small ΔCp values (0.020–0.073 J g^−1^ °C^−1^) corroborate that this transition was exclusive to the amorphous xanthan fraction, with no major structural rearrangement.

A minor enthalpic relaxation peak was observed immediately after the glass transition in all samples. The peak transition was recorded between 148.03 and 155.57 °C, which gave a range of enthalpy values from 2.46 to 5.68 J/g for this thermal event, which is reported as the systematic process representing the progressive recovery of structural imperfections in non-equilibrium amorphous polymer prior to reaching thermal equilibrium following glass transition (Hodge, 1994 [56]). The pronounced difference in the magnitude of relaxation enthalpy that was observed for the Sukkari xanthan samples indicates a much larger extent of complex molecular rearrangement during the heating period, whereas conversely, the low enthalpy values recorded for Barhi suggest a more thermodynamically relaxed molecular structure before initial thermal analysis. The reported differences in enthalpic relaxation for the variety of samples tested could be due to fundamental differences between the overall molecular weight, drying history, spatial orientation of polymer chains, and the primary hydrogen bonding network established during the fermentation and purification steps. Another observation is made at around a peak temperature of 182–198 °C, which represents an endothermic phenomenon but certainly not a melting point since Xanthan is a high-molecular-weight polysaccharide with much intra-and intermolecular hydrogen bonding, and does not have a sharp melting point like low-molecular-weight crystalline materials. It better represents a combination of physical transformations and the early product degradation processes, such as volatile loss in the form of vaporization, disruption of hydrogen bonds, and initial chain scission. This interpretation is further substantiated by the TGA results. Initial mass loss began at around 160 °C with the main degradation step occurring at around 287–297 °C, as shown by its maximum rate of loss. That latter event is the main thermal decomposition of the xanthan gum matrix and corresponds to an advanced depolymerization of polymer-chains. Accordingly, the DSC and TGA results correlate with each other as complementary: the endothermic event of DSC between 182 and 198 °C illustrates a process of complex thermal change in conjunction with initial degradation stages while TGA peaks at 287–297 °C represent the main degradation process.

#### 3.6.1. Steady Shear Rheology

**Figure 4:**
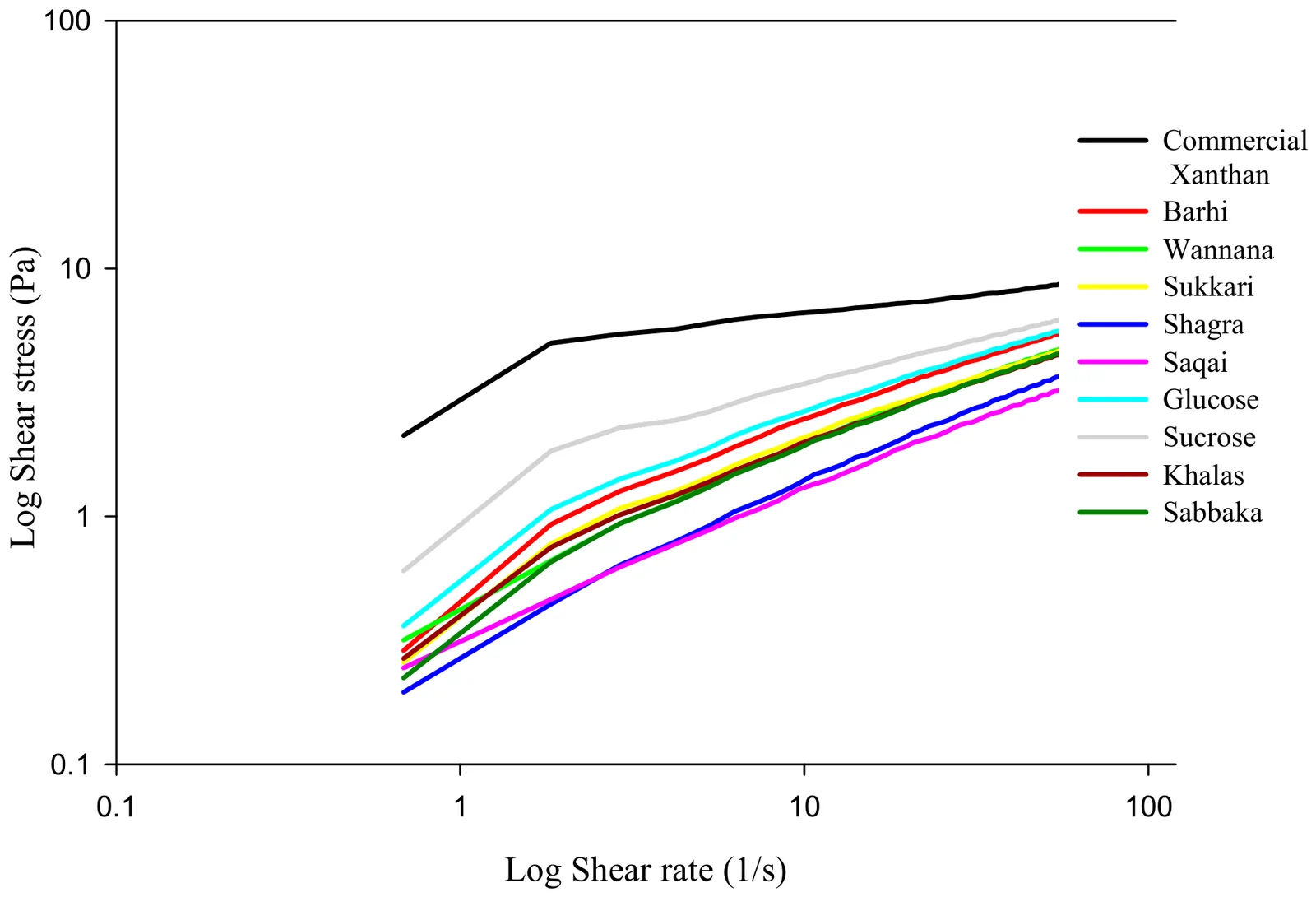
Steady shear rheological behavior of xanthan gum (0.5% solutions at 20 °C) produced from different date cultivars.

**Figure 5:**
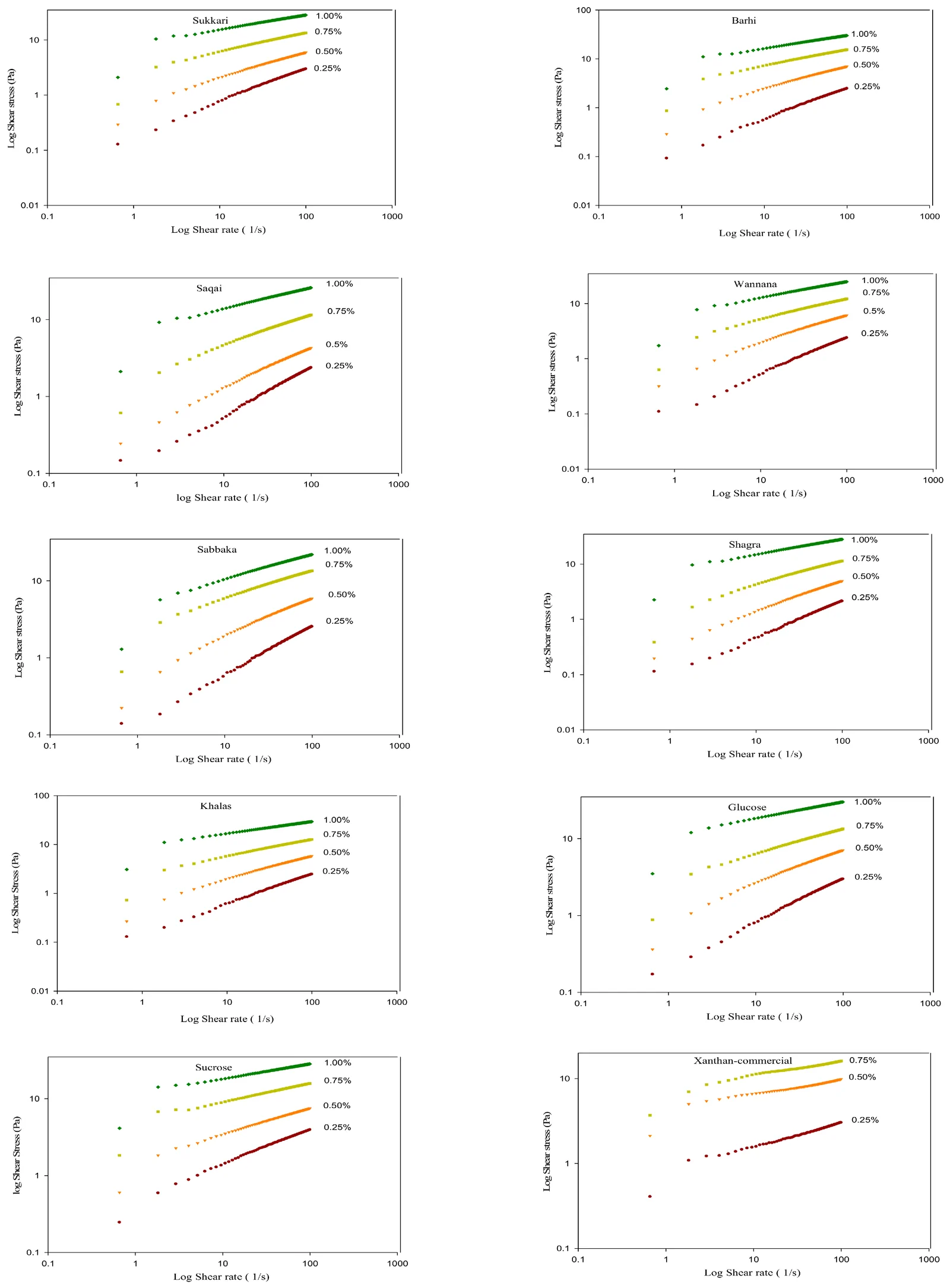
Shear rate vs. shear stress curves of xanthan gum samples as a function of concentration at 20 °C.

Steady shear rheological behavior of the different xanthan samples was determined at different concentrations and temperatures. The data was fitted to the power law model τ = Kγ^n^, where the K value represents the consistency index/apparent viscosity (Pa·s^n^) while flow behaviors index (deviation from the Newtonian flow) is denoted by n. The values of R^2^ presented in Table 7 explain that the model was best-fit for all of the study samples, concentrations and temperatures. The power law model is used successfully by researchers to explain the flow behavior of different systems involving xanthan gum [42,57,58]. The shear stress–shear rate curves shown in Figure 4 and Figure 5 provide further support to the power law analysis. All the curves showed a nonlinear upward trend, which is typical for pseudoplastic materials, with the continuous increase in the shear stress with the shear rate and gradual decrease in the rate of increase. With the increase in XG concentrations, the curves became steeper and the values of shear stress were significantly higher, which corresponded to the increases in K. Commercial xanthan always caused the highest shear stresses, but all the xanthan samples obtained in the laboratory showed similar rheological behaviors, with differences mainly due to the fermentation substrate.

##### Effect of Gum Concentration on Consistency Index

One of the most prominent trends observed was a substantial increase in the consistency index (*K*) with increasing xanthan gum concentration (Figure 4 and Figure 5). The *K* values of the xanthan gums produced in the laboratory at 20 °C increased from 0.10–0.52 Pa·s^n^ at 0.25% to 4.96–11.34 Pa·s^n^ at 1.0%, a nearly 20- to 50-fold increase depending on the substrate. Similarly, the viscosity of glucose xanthan increased from 0.24 to 11.01 Pa·s^n^ and sucrose xanthan increased from 0.52 to 11.34 Pa·s^n^ in the same concentration range. The highest consistency was observed for commercial xanthan gum up to 0.75%, which increased from 0.78 Pa·s^n^ at 0.25% to 7.37 Pa·s^n^ at 0.75%. However, measurements at 1.0% were not possible because of the very high viscosity of the commercial xanthan gum sample. The progressive overlap and the entanglement of the xanthan gum are reported to be responsible for the increase in the consistency coefficient and decrease in the flow behavior index (shear thinning) of the xanthan solutions with increasing concentration [59]. At low polymer concentrations xanthan molecules are quite isolated and move independently. With increasing concentration intermolecular associations, hydrogen bonding and chain entanglements increase, resulting in a stronger three-dimensional network resisting deformation during shear. Alam, Malakar, Pant, Dar and Nanda [58] reported that xanthan gum possesses excellent thickening efficiency and better shear thinning behavior at low concentrations when compared with guar gum. Increasing gum concentrations in the formulations increased both apparent viscosities and the consistency coefficient (*K*) [60].

##### Effect of Temperature on Consistency Coefficient and Flow Behavior Index

Temperature had a reverse effect on solution consistency (Table 7). In all samples, increasing temperature from 20 °C to 40 °C resulted in a consistent decrease in *K* values. For example, Sukkari xanthan (1%) was reduced from 8.17 to 5.32 Pa·s^n^, Khalas xanthan from 9.47 to 5.69 Pa·s^n^, glucose-based xanthan from 11.01 to 7.95 Pa·s^n^ and sucrose-based xanthan from 11.34 to 8.62 Pa·s^n^. Commercial xanthan showed a gradual reduction from 7.37 to 6.30 Pa·s^n^ at 0.75% concentration. The decrease in consistency with the increase in temperature is attributed to the thermal degradation of intermolecular forces in the xanthan gum network [61]. An increase in temperature increases the mobility of molecules, which influences the hydrogen bond, chain flexibility and flow resistance [62]. Although the moderate reduction in *K* happened with the increase in the temperatures, the stability of xanthan gum is solid enough to make it a suitable candidate for foods with moderate heat processing.

The flow behavior index (*n*) was found to be less than 1 for all the samples at all the experimental conditions confirming that all the xanthan gum solutions behaved as non-Newtonian pseudoplastic (shear-thinning) fluids. Across a wide range of hydrocolloids, xanthan gum exhibits significant shear thinning behavior [42,63,64]. The n values for 0.25% concentration were in the range of 0.29 to 0.70 while for 1.0% concentration n values decreased to a range of 0.20 to 0.32 depending on the substrate. All formulations have a flow behavior index (*n*) < 1.0, indicating a shear thinning behavior. The decrease in n with increasing concentration indicates that xanthan gum solutions were more shear-thinning as polymer concentration increased. For example, Sukkari xanthan *n* value decreased from 0.58 at 0.25% to 0.27 at 1.0%, Barhi xanthan from 0.61 to 0.27, glucose-derived xanthan from 0.56 to 0.22 and sucrose-derived xanthan from 0.44 to 0.20 in the same concentration range. The gum concentration directly impacts its flow behavior index, keeping it within the non-Newtonian shear thinning range [60,65]. It is reported that shear-thinning behavior of the hydrocolloid gums is highly desirable in food systems as it provides high viscosity under stationary conditions for product stability, but a decrease in viscosity during pumping, mixing, swallowing or filling operations eases the processing [62,66,67]. Concentration decreased n, but flow behavior index slightly increased with the increase in temperature. For example, the n value of Khalas xanthan increased from 0.25 at 20 °C to 0.31 at 40 °C, glucose-derived xanthan increased from 0.22 to 0.25 and sucrose-derived xanthan increased from 0.20 to 0.23. The slight increase indicates that the xanthan gum solutions were less shear-thinning at elevated temperatures, as the higher thermal energy causes partial breakdown of intermolecular associations before shear is applied.

##### Effect of Substrates

The difference in the fermentation media substrates also affected the rheological properties of the xanthan gum. Xanthan gum produced from the date cultivars like Barhi, Khalas, Shagra and Sukkari had higher consistency indices. On the other hand, Sabbaka represented the lower k values when compared at higher concentrations. Although traditionally glucose and sucrose are used as the carbon sources to produce xanthan gum for industrial production, many researchers have identified other potential sources, e.g., olive pomace, grape pomace, *Melaleuca alternifolia*, cheese whey, milk permeate, sugarcane bagasse, and date juice, and have harvested xanthan gum with acceptable rheological properties [36,37,38]. The xanthan produced in the current study from different date substrates had similar rheological behavior as those produced with commercial substrates like glucose and sucrose. This could be a potential benefit of utilizing the low-cost raw materials for sustainable production of xanthan gum using biotechnology. The relative differences observed among the xanthan from the different substrates could be attributed to the polymer chain length, purification, molecular weight and pyruvate and acetyl substitution in the polymer network.

#### 3.6.2. Dynamic Rheology

**Figure 6:**
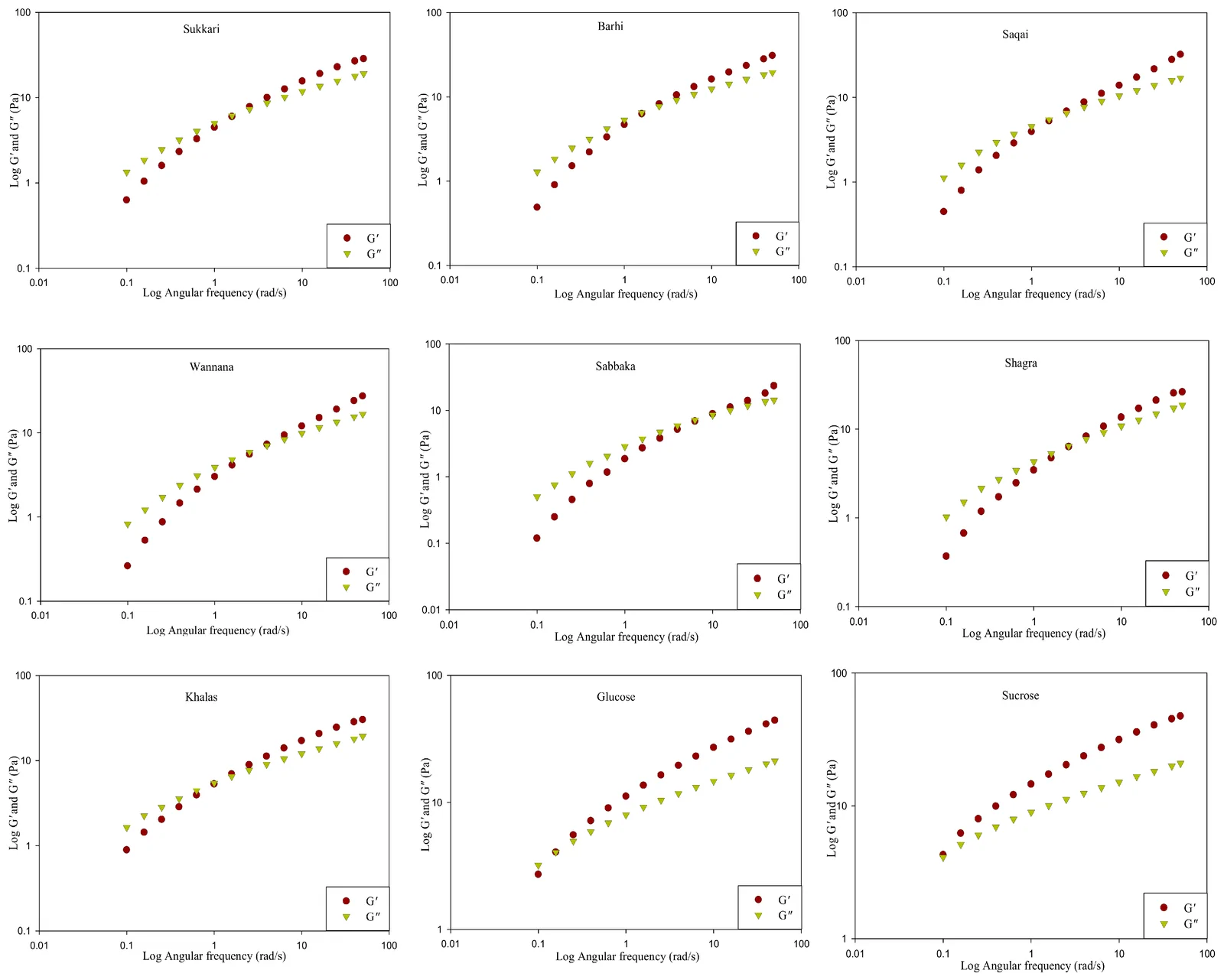
Frequency sweep dependency of storage (G′) and loss modulus (G″) of xanthan gum solution (1%) at 25 °C.

The data presented in Figure 6 reflects the dynamic viscoelastic behavior of the 1% xanthan solutions in a constant strain (5%) in the linear viscoelastic region. The frequency sweep test was performed from 0.01 rad/s to 50 rad/s at 25 °C. As the data indicates, the overall slope of storage modulus (G′) was higher than loss modulus (G″). The value of G″ was higher than that of G′ at low frequency and the phenomenon reversed at higher frequency ranges, with crossover happening in almost all the studied lab-grown xanthan samples. For the regions at a lower frequency before the crossover point, the samples had viscous-like behavior (G″/G′), while higher frequency regions (after crossover) resulted in G′/G”, indicative of solutions solid/gel-like behavior. The studies on commercial xanthan gums represented more elastic properties (G ′/G″) with no crossovers [68,69,70]. On the other hand, the crossover in produced samples is likely due to variations in molecular weight distribution and substitution patterns when compared to commercial xanthan gum. These factors can result in differences in the chain flexibility, inter- and intramolecular association, and network formation, which consequently create an effect on viscoelastic response. The overall frequency sweep results confirm that the rheological properties of xanthan gum obtained from different date cultivars are similar to the properties of xanthan produced using conventional glucose and sucrose media. The modulus values showed slight quantitative differences but all the samples were characterized by a predominantly elastic response with stable viscoelastic networks. The findings suggest that date juice may be used as an alternative carbon source for xanthan gum biosynthesis without affecting the basic rheological functionality of xanthan gum. The produced xanthan can be used for thickening, suspension stabilization and texture enhancing in food systems.

### 3.7. Activation Energy of the Xanthan Gum Samples

The Arrhenius mathematical model successfully characterized the relationship of temperature dependence of the consistency coefficient (K) for the 0.25% *w*/*v* solutions (From Table 7) of the xanthan gum produced from different substrates. This model yielded R^2^ values in an excellent fitting region of 0.97–0.99. In addition, these consistently high R^2^ values confirm that the complex rheology of each of the xanthan gum samples was strictly consistent with the Arrhenius-type temperature dependence for the studied range of temperatures (20, 30 and 40 °C).

It was observed that Ea values differed from 9.98 to 29.39 kJ/mol among different samples (Table 8). Fluid dynamics and polymer science explain that the activation energy is an essential metric that provides reliable insight into a fluid’s viscoelasticity response to variation in temperature. The elevated Ea values characteristically denote that viscosity drops off sharply with rising thermal gradients while conversely lower Ea values are indicative of a pronounced structural durability/resilience towards temperature-driven changes in viscosity [71]. It is evident from the table that the xanthan from Saqai date syrup medium specifically demonstrated the highest value (29.39 kJ/mol) for activation energy followed by samples derived from Shagra (26.45 kJ/mol), Sabbaka (26.44 kJ/mol) and finally the standard glucose control medium (26.44 kJ/mol). The noticeably larger Ea parameters therefore strongly imply that the xanthan gums in this study had a distinctly greater degree of temperature sensitivity and set up a physical scenario where the fragile intermolecular forces and intricate physical entanglements within the three-dimensional polymer network were much more readily disrupted as ambient temperatures systematically increased. By contrast, lower (21.40 kJ/mol) and much lower (19.53 kJ/mol) thermodynamic values were related to xanthan gums produced from Sukkari and Barhi substrates while intermediate tier of activation energies was recorded for the xanthan gums produced from Khalas, Wannana and standard Sucrose medium (23.90, 23.67 and 23.58 kJ/ mol, respectively). These results strongly indicate that the xanthan gums successfully generated from these two latter substrates were able to retain their intrinsic viscosity better and more consistently under thermal processing, and thus had a distinctly higher degree of intrinsic thermal stability compared to the previously mentioned higher-Ea samples. The activation energies of around 14.7–15 KJ/mol for xanthan gum solutions were also reported by several other researchers [71,72,73,74].

A lower activation energy is an indication of a more stable macromolecular polymer network exhibiting less thermal dependence on dynamic viscosity, which is generally considered to be one of the most important desirable properties for food products that need to undergo continuous thermal processing processes or hot filling processing. The commercial xanthan gum exhibited the lowest activation energy of only 9.98 kJ/mol. This lower Ea value is very representative of better thermal stability. This very stable rheological behavior is perfectly reasonable and consistent, because commercial xanthan gum production is usually conducted in tightly controlled, highly optimized industrial-scale fermenter environments. As a result, these industrial products may inherently have a better structural integrity, in addition to specific ratios of acetyl or pyruvate side-chain substitutions which consequently work together towards the attainment of the improved temperature-induced viscosity loss [31].

Additionally, the pre-exponential factor (K_0_) showed higher variations across the variety of samples analyzed. The xanthan gums produced specifically in a laboratory displayed natural logarithmic ln(K_0_) values that ranged from −14.055 to −9.926, corresponding mathematically to absolute K_0_ values between 7.89 × 10^−7^ and 4.90 ×10^−5^ Pa·s^I^. Conversely, the K_0_ for the commercial xanthan gum was approximately and significantly much higher in magnitude (1.32 × 10^−2^ Pa·s^n^). The large numerical differences reflect directly the structural differences residing in their very inherent intrinsic rheological properties of the individual xanthan molecules themselves, and additionally they contribute to determining how specifically varied carbon sources employed during biological fermentation have a significant quantifiable impact upon both polymer nano architecture as well as macroscopic flow behavior. Previous studies have demonstrated that the choice of fermentation substrate can alter the molecular weight, chain conformation and degree of substitution of functional side-chains, ultimately affecting the viscosity and temperature-dependent behavior of the xanthan [14,34,72].

## 4. Conclusions

This study demonstrated that low-quality dates from seven Saudi cultivars can be successfully upcycled into food-grade xanthan gum through fermentation with *Xanthomonas campestris*. Date juice proved to be a compositionally rich, monosaccharide-dominated substrate that delivered gum yields of 5.60–7.77 g L^−1^. The Barhi and Saqai cultivars out-yield both the glucose and sucrose controls. FTIR analysis confirmed that the primary structure of the date-derived gums was identical to that of the commercial standard. At the same time, thermogravimetric analysis revealed superior thermal stability, evidenced by higher char residues at 500 °C. The date-based powders were darker than the commercial reference due to residual pigments and Maillard products, which is considered a limitation readily addressable by a simple decolorization step. Nevertheless, their rheological behavior was fully comparable, exhibiting pseudoplastic, shear-thinning characteristics and the concentration- and temperature-dependence expected of high-quality xanthan gum.

Taken together, these findings establish low-quality Saudi dates as a technically robust and cost-effective alternative to conventional refined-sugar substrates. It offers a viable route to valorize date-processing waste while supporting local, circular-economy production of a commercially important biopolymer for the food, pharmaceutical, and allied industries. Future work should focus on optimizing the fermentation parameters and the downstream purification at pilot scale, and on quantifying the acetyl and pyruvate substitution levels that underpin the functional differences observed among the cultivars.

## Figures and Tables

**Table 1:** Composition of date juices used as a substrate for xanthan gum production.

Substrate	Brix°	Fructose %	Glucose %	Sucrose %
Sukkari	17	4.69	5.00	6.91
Barhi	16.5	8.80	8.83	0.00
Saqai	16	8.24	8.63	0.06
Wannana	16	8.51	8.84	0.00
Sabbaka	16	8.83	8.82	0.00
Shagra	14	7.91	8.02	0.12
Khalas	12.5	8.05	8.69	0.03

**Table 2:** Composition and yield of xanthan gum.

Substrate	Raw Yield/Total Solids (g/L)	Calculated Polymer Yield (g/L)	Moisture (%)	Ash (%)	Protein (%)
Sukkari	7.25	5.62	7.98 ± 0.29 ^c^	13.57 ± 0.10 ^cd^	0.88 ± 0.15 ^cd^
Barhi	7.77	6.10	8.17 ± 0.20 ^c^	12.60 ± 0.13 ^d^	0.70 ± 0.12 ^de^
Saqai	7.58	5.81	7.61 ± 0.28 ^c^	14.44 ± 0.18 ^bc^	1.36 ± 0.08 ^ab^
Wannana	6.45	4.86	7.61 ± 0.44 ^c^	15.59 ± 0.15 ^ab^	1.46 ± 0.11 ^a^
Sabbaka	7.22	5.33	8.08 ± 0.46 ^c^	16.51 ± 0.85 ^a^	1.54 ± 0.06 ^a^
Shagra	6.97	5.22	7.69 ± 0.46 ^c^	16.31 ± 0.73 ^a^	1.08 ± 0.14 ^bc^
Khalas	5.60	4.17	11.24 ± 0.31 ^a^	13.58 ± 0.29 ^cd^	0.68 ± 0.06 ^de^
Glucose	6.68	4.93	10.91 ± 0.39 ^ab^	14.91 ± 0.55 ^abc^	0.39 ± 0.02 ^ef^
Sucrose	6.23	4.77	7.90 ± 0.29 ^c^	15.09 ± 0.51 ^abc^	0.45 ± 0.09 ^ef^
Commercial	-	-	10.37 ± 0.08 ^b^	7.52 ± 0.77 ^e^	0.17 ± 0.03 ^f^

Means carrying different letters in a column are significantly different (*p* ≤ 5) from each other.

**Table 3:** Color parameters of xanthan gum samples from different substrates.

	L*	A*	B*	ΔE	WI
Sukkari	71.00 ± 0.60 ^c^	5.65 ± 0.07 ^d^	21.71 ± 0.28 ^c^	17.74	63.34
Barhi	67.81 ± 0.13 ^d^	4.00 ± 0.09 ^f^	19.13 ± 0.25 ^d^	24.85	62.34
Saqai	62.30 ± 1.66 ^fg^	6.95 ± 0.44 ^b^	23.71 ± 0.80 ^b^	23.26	54.92
Wannana	63.05 ± 0.54 ^ef^	5.31 ± 0.24 ^d^	22.15 ± 0.56 ^c^	22.53	56.59
Sabbaka	63.93 ± 1.07 ^e^	5.63 ± 0.26 ^d^	22.21 ± 0.33 ^c^	25.00	57.27
Shagra	61.09 ± 0.47 ^h^	6.10 ± 0.10 ^c^	21.04 ± 0.24 ^c^	21.57	55.35
Khalas	67.23 ± 1.40 ^d^	7.86 ± 0.28 ^a^	25.44 ± 1.61 ^a^	17.37	57.77
Glucose	69.93 ± 0.82 ^c^	4.81 ± 0.15 ^e^	25.04 ± 0.33 ^a^	10.32	60.58
Sucrose	82.81 ± 0.58 ^b^	1.77 ± 0.10 ^g^	25.53 ± 1.02 ^a^	16.06	69.17
Commercial	84.71 ± 0.40 ^a^	−0.02 ± 0.02 ^h^	15.55 ± 0.28 ^e^	18.21	78.19

Means carrying different letters in a column are significantly different (*p* ≤ 5) from each other.

**Table 4:** FTIR spectra bands position.

	-C-O	C-O-C, C-O	-COO-, C-H, C-O	COO	-C=O	-CH	-OH
	Sugar Ring Vibration	C–O–C and C–O Stretching	COO^−^ Symmetric Stretching, C–H Bending, C–O Stretching	COO^−^ ASYMMETRIC Stretching	Vibration of the Carbonyl (C=O)	Axial DEFORMATION of -CH2	Axial Deformation of -OH
Sukkari	784	1018	1401-1242	1603	1714	2877	3220
Barhi	783	1018	1403-1241	1603	1714	2888	3197
Saqai	785	1017	1404-1240	1603	1714	2875	3193
Wannana	783	1018	1405-1240	1603	1714	2866	3232
Sabbaka	784	1019	1404-1241	1604	1714	2901	3192
Shagra	788	1016	1402-1238	1600	1714	2897	3200
Khalas	789	1014	1403-1239	1603	1715	2897	3198
Glucose	783	1016	1402-1241	1602	1715	2876	3234
Sucrose	782	1017	1403-1240	1602	1715	2880	3213
Xanthan-Commercial	782	1019	1403-1240	1600	1714	2882	3234

**Table 5:** Thermal degradation parameters of the xanthan gum samples.

	Decomposition Stage 1			Decomposition Stage 2				
	Temp. Range (°C)	DTG Maximum (°C)	%wt Loss	Temp. Range (°C)	DTG Maximum (°C)	%wt Loss	Total Weight Loss (1 and 2)	Residue at 500 °C
Sukkari	35–169.41	81.13 ± 0.34 ^b^	7.98 ± 0.29 ^c^	169.41–389.05	293.46 ± 0.33 ^d^	29.94 ± 0.25 ^ef^	37.93 ± 0.34 ^f^	51.51 ± 0.34 ^c^
Barhi	35–164.38	81.53 ± 0.06 ^b^	8.17 ± 0.20 ^c^	164.38–393.99	294.86 ± 1.02 ^c^	30.07 ± 0.23 ^de^	38.25 ± 0.03 ^ef^	52.15 ± 0.55 ^bc^
Saqai	35–166.24	79.07 ± 0.67 ^de^	7.61 ± 0.28 ^c^	166.24–407.76	294.18 ± 0.34 ^cd^	30.95 ± 0.37 ^cd^	38.56 ± 0.12 ^e^	49.79 ± 0.67 ^d^
Wannana	35–160.72	78.46 ± 1.08 ^e^	7.61 ± 0.44 ^c^	160.72–397.09	295.29 ± 0.69 ^c^	33.92 ± 0.47 ^c^	41.53 ± 0.14 ^b^	54.05 ± 0.30 ^a^
Sabbaka	35–165.03	80.01 ± 0.07 ^cd^	8.08 ± 0.46 ^c^	165.03–399.34	296.67 ± 0.89 ^ab^	29.08 ± 0.84 ^fg^	37.16 ± 0.57 ^g^	53.64 ± 0.53 ^a^
Shagra	35–164.41	80.82 ± 0.06 ^bc^	7.69 ± 0.46 ^c^	164.41–395.15	294.84 ± 0.43 ^c^	30.33 ± 0.70 ^cde^	38.02 ± 0.09 ^f^	52.76 ± 0.09 ^b^
Khalas	35–167.83	83.02 ± 0.71 ^a^	11.24 ± 0.31 ^a^	167.76–396.24	295.47 ± 0.14 ^bc^	28.72 ± 0.30 ^g^	39.95 ± 0.09 ^c^	48.93 ± 0.17 ^e^
Glucose	35–172.59	82.87 ± 0.34 ^a^	10.91 ± 0.39 ^ab^	172.59–391.31	296.70 ± 0.34 ^ab^	27.26 ± 0.41 ^h^	38.17 ± 0.10 ^ef^	50.09 ± 0.24 ^d^
Sucrose	35–164.25	76.04 ± 0.12 ^f^	7.90 ± 0.29 ^c^	164.24–396.28	297.90 ± 0.66 ^a^	31.18 ± 0.31 ^c^	39.08 ± 0.05 ^d^	52.18 ± 0.40 ^bc^
Commercial	35–190.74	78.94 ± 0.28 ^e^	10.37 ± 0.08 ^b^	190.74–380.60	288.36 ± 0.78 ^e^	42.89 ± 0.32 ^a^	53.26 ± 0.27 ^a^	33.49 ± 0.26 ^f^

Means carrying different letters in a column are significantly different (*p* ≤ 5) from each other.

**Table 6:** DSC parameters of the xanthan gum samples.

	Peak 1 (Loss of Free Water)		Peak 2 (Loss of Bound Water)		Glass Transition		Peak 3 (Enthalpic Relaxation)		Peak 4 (Degradation)	
Xanthan Gum Samples	T_p_ (°C)	∆H (J/g)	T_p_ (°C)	∆H (J/g)	Tg (Midpoint) (°C)	∆Cp	T_p_ (°C)	∆ H (J/g)	T_d_ (°C)	∆_d_ H (J/g)
Sukkari	65.29 ± 0.86 ^b^	1.05 ± 0.08 ^cd^	111.97 ± 1.43 ^a^	8.56 ± 1.11 ^d^	139.16 ± 2.14 ^bcd^	0.040 ± 0.005 ^cd^	153.51 ± 0.68 ^ab^	5.68 ± 0.48 ^a^	184.05 ± 1.32 ^def^	157.73 ± 2.72 ^d^
Barhi	62.96 ± 0.38 ^c^	0.98 ± 0.02 ^cd^	110.48 ± 0.61 ^abc^	9.01 ± 0.63 ^cd^	137.28 ± 0.70 ^d^	0.020 ± 0.003 ^f^	148.78 ± 0.88 ^de^	2.46 ± 0.15 ^e^	185.67 ± 0.62 ^cdef^	136.47 ± 3.30 ^f^
Saqai	63.19 ± 0.36 ^c^	1.41 ± 0.26 ^b^	109.71 ± 0.64 ^bcde^	7.76 ± 0.27 ^de^	141.37 ± 0.35 ^bc^	0.043 ± 0.006 ^bcd^	155.57 ± 1.58 ^a^	3.86 ± 0.20 ^cd^	191.47 ± 1.57 ^b^	145.26 ± 2.61 ^e^
Wannana	65.88 ± 1.27 ^bc^	1.27 ± 0.09 ^bc^	109.96 ± 0.22 ^bcd^	9.79 ± 0.45 ^b^	144.96 ± 0.31 ^a^	0.073 ± 0.005 ^a^	155.48 ± 0.90 ^a^	4.57 ± 0.31 ^bc^	187.16 ± 1.52 ^cd^	160.46 ± 2.43 ^cd^
Sabbaka	63.09 ± 0.26 ^c^	1.49 ± 0.08 ^b^	109.27 ± 0.97 ^cde^	9.32 ± 0.66 ^cd^	141.87 ± 1.97 ^b^	0.031 ± 0.002 ^e^	152.02 ± 1.10 ^bc^	4.87 ± 0.71 ^ab^	198.86 ± 1.25 ^a^	134.65 ± 4.26 ^f^
Shagra	62.82 ± 0.50 ^c^	0.77 ± 0.09 ^de^	111.26 ± 0.83 ^ab^	10.99 ± 0.51 ^a^	140.57 ± 0.54 ^bc^	0.032 ± 0.002 ^e^	149.81 ± 0.51 ^cde^	4.41 ± 0.09 ^bc^	188.85 ± 1.12 ^bc^	161.41 ± 4.14 ^cd^
Khalas	63.72 ± 0.12 ^c^	1.92 ± 0.15 ^a^	108.25 ± 0.51 ^e^	9.43 ± 0.75 ^cd^	139.53 ± 0.94 ^bcd^	0.046 ± 0.003 ^bc^	148.03 ± 1.36 ^e^	4.58 ± 0.35 ^bc^	182.91 ± 1.41 ^ef^	172.11 ± 2.32 ^b^
Glucose	63.26 ± 0.57 ^c^	1.27 ± 0.20 ^bc^	108.53 ± 0.89 ^de^	9.54 ± 0.58 ^bc^	140.64 ± 1.04 ^bc^	0.032 ± 0.002 ^e^	152.01 ± 1.54 ^bc^	4.62 ± 0.45 ^bc^	191.52 ± 2.87 ^b^	183.74 ± 3.84 ^a^
Sucrose	63.22 ± 0.07 ^c^	0.65 ± 0.02 ^e^	110.67 ± 1.05 ^abc^	6.55 ± 0.68 ^e^	139.07 ± 2.29 ^cd^	0.037 ± 0.002 ^de^	148.77 ± 0.88 ^de^	4.58 ± 0.65 ^bc^	185.93 ± 2.55 ^cde^	166.95 ± 2.90 ^bc^
Commercial	78.41 ± 0.55 ^a^	1.55 ± 0.19 ^b^	-	-	145.26 ± 0.80 ^a^	0.050 ± 0.006 ^b^	150.71 ± 1.07 ^cd^	3.03 ± 0.34 ^de^	182.19 ± 1.67 ^f^	140.79 ± 1.93 ^ef^

T_p_, peak temperature; ∆H, enthalpy change; T_d_, degradation temperature; **∆_d_** H, enthalpy change in degradation. Means carrying different letters in a column are significantly different (*p* ≤ 5) from each other.

**Table 7:** Power law model parameters of different xanthan gums.

	20 °C			30 °C			40 °C		
	*K*	*n*	R^2^	*K*	*n*	R^2^	*K*	*n*	R^2^
				0.25%					
Sukkari	0.21	0.58	0.991	0.15	0.61	0.990	0.12	0.65	0.994
Barhi	0.15	0.61	0.992	0.11	0.64	0.994	0.09	0.67	0.994
Saqai	0.13	0.62	0.989	0.10	0.65	0.993	0.06	0.70	0.993
Wannana	0.13	0.64	0.993	0.09	0.67	0.994	0.07	0.71	0.995
Sabbaka	0.14	0.63	0.994	0.10	0.66	0.990	0.07	0.71	0.990
Shagra	0.10	0.66	0.994	0.07	0.69	0.991	0.05	0.73	0.995
Khalas	0.15	0.60	0.993	0.12	0.63	0.989	0.08	0.67	0.994
Glucose	0.24	0.56	0.987	0.17	0.59	0.994	0.12	0.64	0.995
Sucrose	0.52	0.44	0.992	0.40	0.47	0.995	0.28	0.52	0.993
Commercial	0.78	0.29	0.994	0.71	0.30	0.994	0.60	0.31	0.992
				0.5%					
Sukkari	0.75	0.45	0.991	0.58	0.48	0.991	0.45	0.51	0.992
Barhi	0.90	0.45	0.986	0.70	0.48	0.994	0.46	0.53	0.991
Saqai	0.49	0.51	0.992	0.36	0.55	0.987	0.26	0.59	0.990
Wannana	0.68	0.48	0.993	0.49	0.52	0.989	0.31	0.58	0.994
Sabbaka	0.66	0.48	0.995	0.49	0.51	0.990	0.31	0.58	0.994
Shagra	0.42	0.54	0.993	0.29	0.58	0.994	0.18	0.64	0.993
Khalas	0.69	0.46	0.990	0.51	0.50	0.990	0.33	0.55	0.994
Glucose	1.02	0.42	0.988	0.80	0.45	0.991	0.55	0.51	0.992
Sucrose	1.56	0.34	0.987	1.28	1.37	0.993	1.06	0.39	0.991
Commercial	4.25	0.17	0.992	4.14	0.18	0.994	4.05	0.20	0.991
				0.75%					
Sukkari	2.80	0.34	0.99	2.35	0.36	0.990	1.86	0.39	0.991
Barhi	3.38	0.33	0.990	2.80	0.36	0.988	2.11	0.39	0.990
Saqai	1.92	0.39	0.964	1.49	0.42	0.989	0.98	0.48	0.992
Wannana	2.26	0.37	0.994	1.80	0.40	0.993	1.36	0.44	0.991
Sabbaka	2.61	0.36	0.993	1.98	0.39	0.995	1.28	0.46	0.995
Shagra	1.64	0.42	0.992	1.17	0.47	0.994	0.74	0.52	0.993
Khalas	2.61	0.35	0.994	2.20	0.36	0.991	1.57	0.41	0.992
Glucose	2.99	0.33	0.988	2.54	0.34	0.988	2.09	0.37	0.994
Sucrose	5.06	0.25	0.989	4.71	0.26	0.987	3.94	0.28	0.995
Commercial	7.37	0.20	0.993	6.83	0.21	0.984	6.30	0.23	0.995
				1%					
Sukkari	8.17	0.27	0.982	6.73	0.29	0.994	5.32	0.32	0.994
Barhi	8.65	0.27	0.983	7.01	0.29	0.991	5.36	0.33	0.993
Saqai	7.31	0.28	0.991	6.09	0.30	0.994	4.83	0.32	0.992
Wannana	6.44	0.29	0.993	5.27	0.31	0.989	4.04	0.35	0.990
Sabbaka	4.96	0.32	0.986	4.17	0.34	0.992	3.08	0.38	0.989
Shagra	7.77	0.28	0.990	6.49	0.29	0.990	4.81	0.34	0.988
Khalas	9.47	0.25	0.983	7.11	0.29	0.988	5.69	0.31	0.99
Glucose	11.01	0.22	0.974	9.58	0.23	0.983	7.95	0.25	0.98
Sucrose	11.34	0.20	0.963	10.64	0.21	0.984	8.62	0.23	0.98
Commercial	-	-	-	-	-	-	-	-	-

*K* = (Pa·s) indices are obtained by fitting the data to power law τ = *K*γ·*^n^* and *n* = flow behavior index (dimensionless). This table was used for Arrhenius equation.

**Table 8:** Activation energy parameters of xanthan gum solutions (0.25%) produced from different substrates.

Varieties	Regression Equation	E_a_ (KJ/mol)	ln(K_0_)	K_0_ (Pa s^n^)	R^2^
Sukkari	lnK = −10.356 + 2573.806(1/T)	21.4	−10.356	3.18 × 10^−5^	0.994
Barhi	lnK = −9.926 + 2349.249(1/T)	19.53	−9.926	4.90 × 10^−5^	0.994
Saqai	lnK = −14.055 + 3535.166(1/T)	29.39	−14.055	7.89 × 10^−7^	0.995
Wannana	lnK = −11.766 + 2846.504(1/T)	23.67	−11.766	7.77 × 10^−6^	0.993
Sabbaka	lnK = −12.806 + 3179.613(1/T)	26.44	−12.806	2.74 × 10^−6^	0.994
Shagra	lnK = −13.154 + 3181.197(1/T)	26.45	−13.154	1.94 × 10^−6^	0.992
Khalas	lnK = −11.672 + 2875.033(1/T)	23.9	−11.672	8.54 × 10^−6^	0.974
Glucose	lnK = −12.272 + 3180.460(1/T)	26.44	−12.272	4.68 × 10^−6^	0.993
Sucrose	lnK = −10.309 + 2835.879(1/T)	23.58	−10.309	3.33 × 10^−5^	0.992
Commercial	lnK = −4.328 + 1199.771(1/T)	9.98	−4.328	1.32 × 10^−2^	0.974

K_0_ (Pa s^n^) = is the frequency factor at a reference temperature (20, 30 and 40 °C); E_a_ = activation energy (KJ/mol K^−1^).

## Data Availability

The publication includes the original contributions made in the study. For any additional inquiries, please contact the corresponding author.
